# Sequencing and *de novo* assembly of a *Dahlia* hybrid cultivar transcriptome

**DOI:** 10.3389/fpls.2014.00340

**Published:** 2014-07-17

**Authors:** Erik M. Lehnert, Virginia Walbot

**Affiliations:** ^1^Department of Genetics, Stanford University School of MedicineStanford, CA, USA; ^2^Department of Medical Microbiology and Immunology, University of Wisconsin School of MedicineMadison, WI, USA; ^3^Department of Biology, Stanford UniversityStanford, CA, USA

**Keywords:** *Dahlia*, next-generation sequencing, *de novo* transcriptome assembly, anthocyanin biosynthesis, floral homeotic factors

## Abstract

*Dahlia variabilis*, with an exceptionally high diversity of floral forms and colors, is a popular flower amongst both commercial growers and hobbyists. Recently, some genetic controls of pigment patterns have been elucidated. These studies have been limited, however, by the lack of comprehensive transcriptomic resources for this species. Here we report the sequencing, assembly, and annotation of the transcriptome of the developing leaves, stems, and floral buds of *D. variabilis*. This resulted in 35,638 contigs, most of which seem to contain the complete coding sequence, and of which 20,881 could be successfully annotated by similarity to UniProt. Furthermore, we conducted a preliminary investigation to identify contigs with expression patterns consistent with tissue-specificity. These results will accelerate research into the genetic controls of pigmentation and floral form of *D. variabilis*.

## Introduction

Horticultural dahlias (*Dahlia variabilis*) are among the most diverse in floral form and colors of all popular garden flowers. Dahlias are also an important contributor to the more than US $100 Billion worldwide flower and potted plant market. Taxonomically dahlias are Compositae, and the center of species diversity is in Mexico and Central America. Likely through cross-breeding of two wild species followed by human selection since 1800 (Gatt et al., 1998), thousands of varieties have been generated by European horticulturists who prized novelty in overall flower size, color, petal number, and petal form.

Garden dahlias are proposed to be recent octoploid derivatives (2N = 64) (Gatt et al., 1998) from crosses between two natural species. Although this high ploidy may have been expected to result in redundancy of genetic factors, loci implicated in flower color have been elucidated and generally behave as diploid factors (Bate-Smith et al., 1955). More recently, genes that are highly expressed in flowers and encoding anthocyanin pathway enzymes have been cloned (Suzuki et al., 2002; Ohno et al., 2011b, 2013) and some enzymes characterized (Yamaguchi et al., 1999; Ogata et al., 2001). Furthermore, a partial reference transcriptome assembly was performed for a comparative genomics study of Compositae, but the majority of contigs did not contain the complete coding sequence (Hodgins et al., 2014).

Given the relatively low cost of deep transcriptome analysis using next-generation DNA sequencing, we wished to determine if reasonable descriptions of messenger RNA diversity could be obtained for leaves, stems, and floral buds of dahlia. The contigs assembled from the transcriptome data are sufficient to propose more than 20,000 likely protein coding genes in this species and can thus serve as a standard for allele comparison among dahlia varieties and for future studies of transposon-mediated variegation in anthocyanin pigmentation and the control of floral form. A cultivar containing anthocyanin in the stems and leaves and flavonoid precursors and anthocyanin in the petals was selected to assess recovery of known pigment factor transcripts as a test of the transcriptome completeness.

## Materials and methods

### Plant growth and RNA isolation

The cultivar “Rio Riata,” originally obtained from Corralitos Gardens (http://www.cgdahlias.com/), was grown outdoors at Stanford University; this variety has 8 orange-red petals with yellow-tips, purple (anthocyanin pigment) stems, and green leaves tinged with purple (http://www.stanford.edu/group/dahlia_genetics/cultivars/rio_riata/rio_riata.htm). Total RNA was extracted from newly emerged stems, leaves, and buds (~0.2–1 cm in diameter) using the RNAqueous-4PCR Kit (Ambion AM1914, Grand Island NY) following the manufacturer's instructions. The RNA-integrity number (RIN) of each sample was determined using an Agilent 2100 Bioanalyzer, Santa Clara CA), and only samples with a RIN ≥ 8 were used. Approximately 1 μg of total RNA was processed (including a poly-A^+^-selection step) from the leaf, stem, and bud extracts using the TruSeq RNA Sample Prep Kit (Illumina FC-122-1001, Hayward CA) following the manufacturer's instructions to produce indexed libraries. The resulting libraries were pooled based on their indices (as described in the kit instructions). Clustering and sequencing were performed by the Stanford Center for Genomics and Personalized Medicine using an Illumina HiSeq 2000 sequencer to generate 101-bp paired-end reads. Accession numbers for reads from bud, leaf, and stem are: SRR1222985, SRR1226613, SRR1226614, respectively.

### Read filtering, transcriptome assembly, and annotation

Transcriptome assembly was performed combining reads from all three tissues. Prior to assembly, the reads were processed as previously described (Lehnert et al., 2014) and outlined here: (1) the first 6 bp from the 5′ end of each read was discarded; (2) reads of <60 bp or containing ≥1 N were discarded; (3) low quality reads were discarded (if >10 of the first 35 bases had quality scores <30); and (4) reads were trimmed to the first position for which a sliding 4-bp window had an average quality-score of <20. The remaining read-pairs were then processed using FLASH (Magoc and Salzberg, 2011) to join reads whose ends overlapped by ≥10 bp with no mismatches. Finally, adapter sequences were removed using cutadapt (Martin, 2011) with default settings.

The processed reads were assembled using a 43-bp *k*-mer with the Velvet/Oases assembler (Velvet version 1.2.09 and Oases version 0.2.08) (Zerbino and Birney, 2008; Schulz et al., 2012) with the default settings. To choose a single representative contig from each “locus” (the Oases term for a connected component in the de Bruijn graph, which presumably consists of alternative transcripts, alleles, and extremely similar paralogs), the script “process_oases_transcripts.py” was used (Yang and Smith, 2013). This script designates as representative the contig with the highest geometric mean *k*-mer coverage that is also at least 30% as long as the longest contig within the locus. Only contigs longer than 300 bp were retained in the resulting set of representative contigs. Contigs were post-processed to remove terminal Ns, and such that internal runs of Ns were fewer than 14 bases in length.

To assign putative functional roles to the representative contigs, they were aligned to the SwissProt protein database and the NCBI Non-Redundant Protein Database (nr) using the blastx program from the standalone BLAST 2.2.25+software suite (Camacho et al., 2009) with an *E*-value cutoff of 1e-5. The Blast2GO software package (Conesa et al., 2005) was used with default settings to assign Gene Ontology (GO) terms and Enzyme codes to the predicted proteins based on their alignments to SwissProt. Blast2GO was also used to identify protein domains by InterProScan (Quevillon et al., 2005), whose associated GO terms were merged with those identified by alignment to SwissProt.

### Expression analysis

The trimmed forward reads were aligned to the representative contigs using bwa (Li and Durbin, 2009). Aligned reads were counted using the samtools (Li et al., 2009; minimum mapping quality 30). The R package DESeq2 (Anders and Huber, 2010) was used to normalize read counts by library size.

## Results

### Sequencing and *de novo* transcriptome assembly

A set of 149,304,876 reads of 101-bp was generated in the initial sequencing run. After trimming and discarding low-quality reads, ~73 million reads from buds, leaves, and stems (see Table 1) were assembled into 53,037 loci containing 122,053 contigs. After removing contigs that fell below the 300-bp length cut-off, choosing representative contigs for each locus, and removing unnecessary Ns (see Materials and Methods), 35,638 representative contigs remained. These ranged in size from 269 bp to 13,886 (see Table 2). These contigs have been deposited in the NCBI TSA (accession # GBDN01000001-GBDN01035638) and can also be found at (http://www.stanford.edu/group/dahlia_genetics/). 20,881 (58.5%) of contigs could be annotated by BLAST alignment to the UniProt protein database. Using these alignments and the results of InterProScan protein motif searches, 122,654 GO terms were assigned to 21,576 (60.5%) of the contigs (see Table 3).

**Table 1:** **Summary of read metrics by tissue of origin**.

	**Total number of reads**	**Total nucleotides (Mb)**	**Average Length (bp)**
Floral (paired)	12,792,456	1118	87
Floral (flash extended^a^)	9,456,389	1264	133
Leaf (paired)	14,541,486	1258	
Leaf (flash extended)	13,742,631	1840	133
Stem (paired)	11,051,702	958	86
Stem (flash extended)	11,455,453	1499	130
Total	73,040,117	7938	–

a *Flash extended reads are paired reads that had 10-bp or more of perfect overlap at the ends of their sequence. These were joined to make longer reads, and are not included in the counts of paired reads (see Materials and Methods)*.

**Table 2:** **Size distribution of the representative contigs**.

	**Representative contigs**	**All contigs**
Number of loci	35,638	53,037
Number of contigs	35,638	122,053
Median contig size (bp)	906	943
Mean contig size (bp)	1166	1209
Minimum contig size (bp)	269	100
Maximum contig size (bp)	13,886	17,090
Total bases in assembly (Mb)	41.6	148

**Table 3:** **Summary of alignments to SwissProt and nr**.

**Number of contigs**	**Number (%) of contigs aligned to SwissProt^a^**	**Number (%^b^) of unique accessions**	**Number (%) of contigs aligned to nr^a^**	**Number (%^b^) of unique accessions**
35,638	20,880 (58.6)	10,603 (50.8)	26,940 (75.6)	21,376 (79.3)

a *Alignments with E-value ≤ 10^−5^*.

b *As % of all alignments*.

### Comparison to known components of anthocyanin biosynthesis in dahlias and identification of additional gene copies

Previous research has identified and sequenced several genes of the dahlia anthocyanin biosynthetic pathway (Suzuki et al., 2002; Ohno et al., 2011a). To investigate assembly quality, we compared these previously sequenced genes to the contigs in our assembly. As shown in Table 4, we identified all the genes previously known to be involved in the synthesis of the anthocyanidins beginning with chalcone synthase. Furthermore, we identified additional copies for several pathway genes by a best-reciprocal-blast approach, thereby expanding the dahlia gene list for the phenylpropanoid pathways. For example, we identified a total of six putative chalcone synthase genes, four more than were previously known. All contigs identified contained the complete coding sequence of the enzyme (as determined by alignment of sequence to the stop and start codon of reference protein), except for one chalcone synthase and for DvIVS, a basic helix-loop-helix transcription factor required for anthocyanin synthesis, in which the cDNA was split between two contigs.

**Table 4:** **Genes putatively required for anthocyanidin synthesis**.

**Putative enzyme**	**Locus # /transcript #**	**Tissue-specificity^a^**	**Previously identified?**
CHS	27/7	All	CHS2^b^
CHS	49578/1	Elevated in stem	CHS1^b^
CHS	7505/1	Decreased in leaf	
CHS	3129/1	All	
CHS	18623/1	Elevated in bud	
CHS	9318/2^c^	Elevated in bud	
CHI	977/3	All	CHI^b^
CHI	2352/8	All	
CHI	13838/4	All	
DvIVS	7738/1; 36303/2^d^	All	DvIVS^c^
FLS	4250/4	Elevated in stem	
F3'H	824/8	All	
F3'H	16120/4	All	
F3'H	18455/5	All	
DFR	44537/3	All	DFR^b^
DFR	23328/5	Decreased in bud	
DFR	11673/3	All	
DFR	27896/1	Decreased in bud	
ANS	45613/2	All	ANS^b^
ANS	27069/1	All	
F3H	22534/2	All	F3H^b^
F3H	33298/1	All	
F3H	39959/2	Decreased in bud	
F3H	43247/1	All	

a *Expressed in all three samples at levels that did not differ enough to be designated as consistent with tissue-specific expression, designated as All*.

b *Ohno et al., 2011b*.

c *Ohno et al., 2011a*.

d *These loci lacked either conserved start or stop codons*.

### Investigating tissue-specific expression

To achieve statistical power in assigning contigs to classes differentially expressed between tissue types, biological replicates would be required. Nonetheless, as a preliminary investigation that might guide future experimental design, we identified contigs with expression patterns consistent with tissue specificity. To do this, we used DESeq to perform a regularized log2 transformation on the read counts; this analysis generates normalized expression values after accounting for the differences in sequencing depth. We assigned a tentative tissue source to a contig by the following procedures: (1) We calculated the standard deviation of expression values across all tissues; (2) we calculated the standard deviation of expression values for each pair of tissues; (3) a contig's expression was classified as elevated or decreased in a specific tissue if the standard deviation of expression values of the pair of other tissues was 3-fold less than the standard deviation of the expression values of all three tissues and the standard deviation of the three tissues expression value was great than 10% the mean expression value. This selected for contigs that showed stable expression in two of three tissues, with a large difference in read counts in the third. Table 4 presents this analysis for the anthocyanin biosynthetic pathway, and Table 5 considers all classes of representative contigs.

**Table 5:** **Tentative tissue specificity of contigs**.

**Expression pattern**	**Number in this class**
Decreased in buds	1407
Elevated in buds	390
Decreased in leaves	2059
Elevated in leaves	1409
Decreased in stems	2690
Elevated in stems	868

This analysis resulted in tentative tissue-specific expression patterns for 8823 contigs (see Table 5). Given the distribution of pigment in Rio Riata, genes essential for anthocyanin synthesis would be expected in the transcriptomes of all three organs, and they were found. Interestingly, while genes for some of the enzymes exhibited similar expression in all three tissues, and all tissues had all the essential components of the pathway for pigment synthesis, some copies (alleles) exhibited patterns consistent with tissue-specific expression (see Table 4). We verified that these results were not affected by reads mapping to multiple contigs, as using only uniquely mapping reads gave nearly identical results. This implies that the apparent diploid nature of dahlia floral pigmentation genes may reflect not only the loss of function of redundant gene-copies, but also evolved tissue-specific expression patterns for some of the redundant genes, a process termed subfunctionalization. As a second test of the classification approach, we investigated the tissue-specificity of contigs annotated as floral homeotic proteins. Of the 22 contigs annotated as similar to floral homeotic proteins, 12 were elevated in buds as expected, while the remainder were classified as lacking tissue specificity (see Table 6).

**Table 6:** **Putative floral homeotic genes in Dahlia**.

**Locus# /transcript#**	**Match to floral homeotic gene**	**Best blast hit accession number**	***e*-Value**	**Read counts in bud**	**Read counts in leaf**	**Read counts in stem**	**Tissue-specificit^a^**
312/8	PMADS2	Q07474	4.47e-71	5697	1	69	Elevated in bud
3477/2	APETALA1	Q41276	9.28e-92	2034	0	22	Elevated in bud
16671/2	AGAMOUS	Q03489	8.38e-119	1192	3	17	Elevated in bud
324/3	AGAMOUS	Q40872	3.30e-128	1163	7	14	Elevated in bud
5269/3	DEFICIENS	P23706	3.68e-96	895	0	18	Elevated in bud
5867/6	DEFICIENS	P23706	1.82e-91	729	2	18	Elevated in bud
3943/2	AGAMOUS	Q03489	9.65e-133	645	41	11	Elevated in bud
643/1	AGAMOUS	Q40872	5.90e-127	637	3	13	Elevated in bud
8582/1	AGAMOUS	Q40872	4.94e-122	245	0	5	Elevated in bud
4783/1	AGAMOUS	Q01540	1.72e-06	157	0	0	Elevated in bud
9368/1	PMADS1	Q07472	1.28e-69	67	6	1	Elevated in bud
9276/1	APETALA2	P47927	1.35e-36	33	0	3	Elevated in bud
7009/1	DEFICIENS	P23706	2.93e-94	1675	27	227	All
1095/3	AGAMOUS	Q40872	4.44e-137	1118	0	54	All
15403/1	APETALA2	P47927	8.53e-111	693	1197	616	All
22399/7	APETALA2	P47927	5.50e-90	350	557	323	All
17401/4	APETALA2	P47927	1.45e-85	214	240	502	All
23847/1	APETALA2	P47927	3.84e-88	140	8	61	All
40009/3	APETALA2	P47927	7.44e-101	135	105	347	All
15550/2	APETALA2	P47927	2.8e-76	98	19	40	All
31911/1	APETALA1	D7KWY6	1.06e-09	30	14	6	All
38896/1	APETALA2	P47927	3.57e-106	16	3	183	All

a *Expressed in all three samples at levels that did not differ enough to be designated as consistent with tissue-specific expression, designated as All*.

## Discussion

Despite the large genome size of *D. variabilis* (9.62 pg; Temsch et al., 2008) and the expectation of two to four paralogs per locus type in an octoploid (Gatt et al., 1998), a paired-end read strategy using next generation sequencing yielded sufficient data to assemble more than 20,000 contigs that could be annotated with GO terms. As flowering plants sequenced to date contain ~25,000–40,000 genes (e.g., Arabidopsis Genome Initiative, 2000; Matsumoto et al., 2005; Amborella Genome Project, 2013) it is likely that the transcriptome data define approximately half of dahlia loci. Genes expressed in seeds, roots, and non-abundant specialized cell types were likely missed as well as genes expressed during biotic or abiotic stress. Given the scanty knowledge about *Dahlia* genes, this new transcriptome resource can serve as a guide in gene identification and cloning, as a standard for comparison among varieties and species, and as a database for identifying genes unique to *Dahlia* and paralogs and orthologs shared with other composites, flowering plants, and all plants. Furthermore, we identified contigs whose expression patterns were consistent with tissue-specificity; future researchers may test our assignments by qPCR or further sequencing. Our method was limited by a lack of biological replicates, which prevents any estimate of the variability of expression of genes within a tissue-type. For this reason, we attempted to be conservative in our calling of specificity and have likely both failed to identify some contigs whose expression is highly enriched in certain tissues, as well as potentially misassigned contigs whose expression is not tissue-specific but is highly variable. However, we believe this resource will be of use for researchers attempting to clone cDNAs or perform *in situ* hybridization to localize specific transcripts. This report indicates that reference transcriptomes for plant species with complex genomes are a feasible and relatively inexpensive method for generating a toolkit for molecular and genetic analysis.

## Author contributions

Erik M. Lehnert performed all of the experiments; Virginia Walbot maintained the dahlias; both authors contributed to design of data collection and analysis and manuscript writing and editing.

### Conflict of interest statement

The authors declare that the research was conducted in the absence of any commercial or financial relationships that could be construed as a potential conflict of interest.
